# Urbanisation and mental health in left-behind children: systematic review and meta-analysis using resilience framework

**DOI:** 10.1038/s41390-025-03894-5

**Published:** 2025-02-05

**Authors:** Kelly Lin, Leona Mak, Jinxuan Cai, Stephen Jiang, Nawaal Fayyaz, Simon Broadley, Jing Sun

**Affiliations:** 1 Rural Health Research Institute, Charles Sturt University, Sydney, NSW Australia; 2 School of Medicine and Dentistry, Griffith University, Brisbane, QLD Australia; 3 Department of Neurology, Gold Coast University Hospital, Southport, QLD Australia; 4 Data Science Institute, University of Technology, Sydney, NSW Australia

## Abstract

**Background:**

Prolonged parental separation at young ages has significant adverse effects on development in left-behind-children (LBC). We aimed to compare mental health status, emotional and behavioural problems, and their association with socioemotional development between LBC and their counterparts.

**Methods:**

Cross-sectional studies comparing LBC and non-LBC published from 2000 onwards were searched. Primary outcomes included depression, anxiety emotional and behavioural problems. Secondary outcomes included loneliness, self-harm, suicide, and risk-related behaviours. Quality of all included articles was assessed by Joanna Briggs Institute (JBI) critical appraisal. Data was analyzed by random model-based effect method.

**Results:**

78 observational studies comprising of 394,308 children aged 2–18 were included. Compared to NLBC, LBC had significantly more depression, anxiety, emotional and behavioural problems, conduct problems, self-harm, loneliness, peer bullying, attempts of smoking and alcohol consumption. Subgroup analyses found that younger LBC between the ages of 6 to 12 were at greater risks of poor mental health, emotional and behavioural problems.

**Conclusion:**

Absent parental care prevents healthy socio-emotional development and hinder the formation of secure attachment. Poor social-emotional development leads to worse emotional resilience against psychological stressors, while LBC residing in rural areas also experience additional risk factors of low household income and poor access to mental health services.

**Impact statement:**

- Prolonged parental separation negatively influences mental health, especially in younger children between the ages 6 to 12.
- Poor social-emotional development in left-behind children is associated with worse emotional resilience against psychological stressors.
- Additional risk factors including residing in rural areas, low household income, and poor access to mental health services predisposes left-behind children to high risks of mental illness.
- Timely support services targeted towards strengthening resilience factor such as learning better emotional and behavioural coping strategies and improving school and peer support to address increased risk of mental health problems are required for current left-behind children.

## Introduction

Left-behind children (LBC) are an expanding group of young individuals in their early childhood or adolescence exposed to long-term, work-related migration of one or both of their parents. Their wellbeing is being increasingly recognised as a prominent social, economic and health problem in many countries.^1^ The global prevalence of LBC is estimated to be more than 100 million.^2^ As a result of rapid urbanization in developing countries, a large number of rural residents from relatively poor, underdeveloped, and geographically isolated areas have chosen to migrate to cities for better job opportunities aimed to improve household income.^3^ Their children are left in the rural settings and their care is entrusted to grandparents, relatives or older siblings due to the high cost of living, schooling in cities and the long working hours.^3^

With improved household income, some studies showed positive effect of parental migration on overall wellbeing and development of LBC.^4–7^ However, a large proportion of epidemiological studies indicated the detriments of early and prolonged separation from parents on the development and mental health of LBC as compared to their counterparts of non-left-behind children (NLBC).

Studies conducted at countries with different income levels and demographic factors have indicated significant heterogeneity in the prevalence of emotional and behavioural problems in children, with an estimate from 5.5% to 18%.^8–10^ Multiple factors are thought to contribute to the development of these problems, including poverty, stressful school and family environments, negative life events, and living in rural areas.^11^ Left-behind children is a unique population vulnerable to emotional and behavioural problems as they are exposed to a range of risk factors associated with poor socioemotional development. Thus, LBC provides us with an important opportunity to assess the effect of aforementioned risk factors on emotional and behavioural problems.

Secure attachment formed between responsive and present caregivers and their child acts as the foundation for healthy socioemotional development.^12^ Children with healthy socioemotional development are more resilient against various psychological stressors that arise in social situations, as they are equipped with effective emotional and behavioural regulation strategies.^13^ In contrast, early and prolonged parental separation predisposes LBC to emotional and behavioural problems as secure attachment cannot be formed between LBC and their parents who are unable to respond timely to their needs.^12,14,15^ Thus, significantly higher prevalence of mental health problems, self-harm and suicidal ideation have been identified LBC than NLBC.^16–19^

To date, several meta-analyses have compared health outcomes, depression, and resilience of LBC to NLBC.^2,20–24^ However, a comprehensive analysis exploring individual parental migration experience and demographic characteristics on the manifestation of emotional and behavioural problems and depression in left-behind children have not yet been conducted. This meta-analysis aimed to compare prevalence of key mental health outcomes, and resilience factors among LBC and NLBC.

## Methods

### Search strategy

The review protocol was registered at Prospero (registration ID: CRD42021224069). A systemic research search conducted between November 2020 to April 2024 was performed using the following databases including MEDLINE, EMBASE, The Cochrane Library, Scopus, PsycINFO, and Web of Science.

Searches were confined to English literature published from 2000 onwards. Key terms searched have been described in appendix 1.

### Inclusion criteria

The criteria used to determine the inclusion of studies in the systematic review and meta-analysis were as follows: (1) Left-behind children were defined as exposure to parental migration by one or both parents for more than six months; (2) migration can be domestic or internal; (3) primary outcomes of interest were depression, anxiety, emotional and behavioural problems; secondary outcomes include loneliness, self-harm, suicide, and health-related behaviours; (4) each study must have a comparison group present; (5) participants were aged 18 and below. Studies were excluded if: (1) children also migrated with parents; (2) in left-behind group, parental migration exposure was defined as anything less than six months; (3) qualitative studies or interventional studies. We specifically excluded studies without a comparison group as the study aimed to compare mental health outcomes between left-behind and non-left behind children. Without a comparison group, differences in prevalence or severity of mental health outcomes could not be determined.

### Data extraction

Title and abstracts of non-duplicated papers using the search strategy were independently screened by two researchers to identify studies that potentially met the inclusion criteria outlined above. Full text of potentially eligible studies was then independently. Study characteristics data were then extracted individually by two researchers for evidence synthesis and assessment of study quality. In case of disagreements on inclusion of studies or data extraction, a third author was consulted. Extracted data included: title, country, publication year, sample size, study design, study population (number of LBC and NLBC), participant demographics (including age, ratio of sex, duration of separation from parents), primary and secondary outcomes, and study quality assessment score.

### Quality assessment

The methodological quality of each study was assessed independently by two researchers using the Joanna Briggs Institute (JBI) critical appraisal. This tool is widely used for cross-sectional studies and meta-analyses in assessing the trustworthiness, relevance, and results of published papers.^25^ If JBI score was less than 5, then the study was considered for exclusion.

### Statistical analysis

Outcome variables were reported as either continuous data or categorical data. Mean difference and effect size for continuous data variables such as depression score were analysed via random effects model. Categorical data outcomes were analysed in the form of risk ratios.

I² was used to assess heterogeneity. Egger regression analysis was conducted to assess publication bias, with further sensitivity analysis if publication bias was identified. Subgroup analyses were also conducted to identify additional factors that may explain any significant associations of poor mental health outcomes in LBC. All subgroup analyses were hypothesis driven. Parental migration status, where two parents have migrated has been significantly associated with worse child mental health outcomes amongst LBC in many previous studies.^14^ In terms of region of residence, LBC residing in rural regions have been found to have worse developmental outcomes due to worse access to healthcare and poor demographic factors such as lower household income and education.^26^ For mean age of child, being left-behind at younger ages have been shown to be a significant risk factor for worse mental health and socio-emotional and behavioural difficulties.^27,28^

## Results

### Selection of studies

We identified 2235 potential records. After removing 1482 duplicate studies, titles and abstracts of the remaining 753 studies were screened and 446 studies were excluded. Full-text reading of 307 studies was assessed for eligibility based on eligibility criteria and JBI score.

**Fig. 1:**
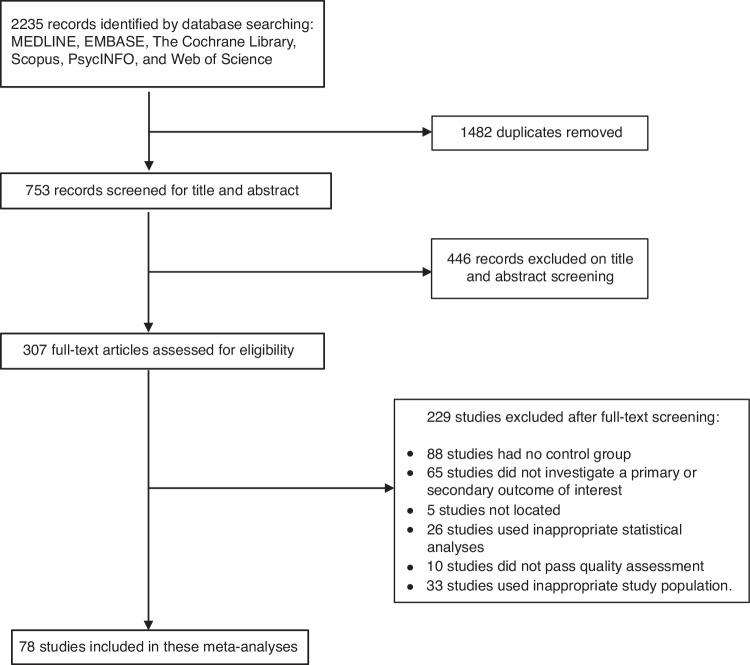
PRISMA flow chart of literature search.

Of these, 229/307 (74.3%) were excluded. Detailed reasoning of excluded studies is presented in Fig. 1. After full-text assessments, 78 studies which met the eligibility criteria were included in this meta-analysis.

### Study characteristics

Full details of the included studies are shown in Table 1. Of the 78 studies, 77 studies conducted research in a single country, while one study recruited participants from multiple countries.^29^ Sixty-nine studies recruited participants living in China, four studies from Indonesia,^30–33^ three in Romania,^34–36^ two in Thailand,^31,37^ one in Philippines^31^ and two in Vietnam.^31^ In terms of study design, 74 studies were cross-sectional in nature, three were cohort studies,^38–40^ and one study was longitudinal.

**Table 1:** Detailed characteristics of studies included in meta-analysis

Study (year)	Country	Sample size	Sex: female, *n* (%)	Mean age	LBC, *n* (%)	Migrated parent	NLBC, *n* (%)	Study type	Data collection format	Mental health assessment tool	Outcome measured	Main caregiver for LBC	Quality assessment result
Adhikari et al.^34^	Thailand	1030	513 (49.8%)		518 (50.3%)	Father (96.5%)	512 (49.7%)	CS	Survey	SDQ	Emotional and behavioural problems, peer bullying, conduct problems	Parent: 494 (95.4%)	Yes
Albin et al.^26^	China	2483	1219 (49.1%)	14	963 (38.8%)		1520 (61.2%)	CS	Survey	SDQ	Emotional and behavioural problems, peer bullying, academic pressure, conduct problems	Parent: 385 (40.6%)	Yes
Akezhuoli et al.^69^	China	2183	956 (43.79%)	13.04	1025 (46.95%)	Both	1158 (53.05%)	CS	Survey	SDQ, Risk Behaviour Surveillance System (YRBSS), Young’s Internat Addiction test for Chinese (YIAT-C)	Conduct problems, smoking, drinking, internet addiction		Yes
Botezat & Pfeiffer^31^	Romania	1421	682 (48.0%)	13.06	279 (19.6%)		1142 (80.4%)	CS	Survey	Depressive symptom questionnaire	Emotional and behavioural problems, peer bullying, academic pressure		Yes
Cai et al.^60^	China	1439	650 (45.17%)	12.4	699 (48.58%)	Both	740 (54.83%)	CS	Survey		Internet addiction, parent-child communication	Grandparents (97.4%)	Yes
Chang et al.^19^	China	13952	6585 (47.2%)	15.22	6034 (43.2%)	Father (54%)	7918 (56.7%)	CS	Survey	University of California Los Angeles Loneliness Scale	Suicide ideation and attempt, peer bullying, loneliness	Parent: 4633 (76.7%)	Yes
Chen & Chan^75^	China	793	380 (48.0%)	12.77	443 (55.9%)		350 (44.1%)	CS	Survey	CES-DC	Depression, peer bullying, conduct problems	Grandparent: 241 (54.4%)	Yes
Chen et al.^98^	China	600	410 (51.0%)	13	227 (37.8%)		373 (62.2%)	CS	Survey	Standardized questionnaire	Delinquency	Parent: 181 (59.5%)	Yes
Cheng et al. (2010)	China	306	150 (49.0%)	12	223 (72.9%)		83 (27.1%)	CS	Survey	SDQ	Psychological well-being		Yes
Chen and Hesketh^99^	China	437	222 (50.08%)	14.85	273 (62.47%)	Both	164 (47.53%)	CS	Survey	Children’s Depression Inventory (CDI-S), Rosenberg Self-esteem Scale (RSES)	Depression, self-esteem, academic	Grandparents (97.4%)	Yes
Cui et al.^84^	China	322	111 (47.8%)	14.4	236 (73.3%)		86 (26.7%)	CS	Survey	Rosenberg self-esteem scale, social support rating scale,	Self-esteem		Yes
Deng et al.^87^	China	436		13.85	149 (34.2%)	Both (100%)	306 (70.2%)	CS	Survey	Youth self-report list, Teacher Support Subscale of MSPSS, Spence Children’s Anxiety Scale	Conduct problem, anxiety, school support		Yes
Faisal & Turnip^27^	Indonesia	629	340 (54.1%)	13.5	359 (57.1%)		270 (42.9%)	CS	Survey	Six-item De Jong Gierveld Loneliness Scale	Loneliness	Parent: 195 (54.3%)	Yes
Fan et al.^42^	China	1274	620 (48.7%)	12.4	629 (49.0%)		645 (51.0%)	CS	Survey	SDQ	Emotional and behavioural problems, conduct problems, peer bullying		Yes
Fu et al.^81^	China	4413	2378 (52.7%)	12.38	1997 (46.5%)	Father (50.2%)	2416 (53.5%)	CS	Survey	CES-DC, multidimensional Anxiety Scale for Children	Suicide ideation and attempt	Parent: 1192 (56.8%)	Yes
Gao^100^	China	2558	1151 (45.0%)	13.8	1126 (44.0%)	Father (68%)	1432 (56.0%)	CS	Survey	Chinese version of Smoking Self-Efficacy Questionnaire	Health behaviours (smoking)	Parent: 1043 (92.6%)	Yes
Gao et al.^71^	China	2986	1451 (48.6%)	14.2	541 (18.1%)		2445 (81.9%)	CS	Survey	Chinese Youth Risk Behaviour Survey	Behavioural and emotional problems, loneliness, suicide intention and attempt, health behaviours (smoking and drinking)	Grandparent: 140 (25.9%)	Yes
Gao et al.^82^	China	8958	4562 (48.9%)	15.54	4716 (52.6%)	Both (70.06%)	4242 (47.4%)	CS	Survey	Social adjustment scale for adolescents	Self-esteem, Parent-child relationship	Parent: 1438 (30.5%)	Yes
Graham & Jordan^28^	Indonesia, Philippines, Thailand, Vietnam	3876			1915 (49.4%)		1961 (50.6%)	CS	Survey	SDQ	Emotional and behavioural problems, conduct problems		Yes
Guang et al.^50^	China	6227	2952 (47.4%)	12.68	4181 (67.1%)	Both (71.3%)	2046 (32.9%)	CS	Survey	CDI	Depression, emotional and behavioural problems, conduct problems, peer bullying, academic pressure	Grandparent: 2798 (66.9%)	Yes
Guo et al.^51^	China	3759	1849 (49.2%)	12.64	1247 (33.2%)	Both (33.9%)	2512 (66.8%)	CS	Survey	CDI	Depression, parent-child relationship, school support, behavioural and emotional problems		Yes
Hu et al.^72^	China	4479	2083 (46.5%)	8.5	1053 (23.5%)		3426 (76.5%)	CS	Survey	Achenbach Child Behaviour Checklist - school-age version	Emotional and behavioural problems		Yes
Huang et al.^101^	China	1363	654 (48.0%)	10.04	608 (44.6%)		755 (55.4%)	CS	Survey	Paediatric Quality of Life Inventory	Health-related quality of life	Grandparent: 479 (78.8%)	Yes
Jia & Tian^77^	China	606	274 (45.2%)	13	324 (53.5%)		282 (46.5%)	CS	Survey	Children’s Loneliness Scale	Loneliness		Yes
Jiang et al.^38^	China	1367	601 (44.0%)	12.2	779 (57.0%)	Both (51.2%)	588 (43.0%)	CS	Interview	Standardized questionnaire	Health behaviours (alcohol), academic pressure	Parent: 380 (48.8%)	Yes
Jin et al.^39^	China	1233	543 (44.0%)		764 (62.0%)		469 (38.0%)	CS	Interview	Standardized questionnaire	Peer support, school support		Yes
Kharel et al. (2021)	Western Nepal	460	326 (52.1%)	14.3	77 (16.7%)	Both (100%)	383 (83.3%)	CS	Survey	SDQ	Emotional and behavioural problem		Yes
Lan & Radin^49^	China	738	403 (54.6%)	15.87	246 (33.3%)		492 (66.7%)	CS	Survey	Chinese Adaptation of the Child Behaviour Checklist	Behavioural problem		Yes
Li et al.^102^	China	3346	1647 (49.2)		1663 (49.7%)		1683 (50.3%)	CS	Survey	Chinese version of the suicidal module from modified MINI Kid 2.0, Olweus bullying questionnaire	Suicide, bullying		Yes
Ling et al.^52^	China	496	225 (45.4%)	11.88	268 (54.0%)	Both (51.1%)	228 (46.0%)	CS	Survey	Youth Self Report, Depression Self-rating Scale for Children, Children’s Loneliness Scale	Depression		Yes
Lu et al.^103^	China	4338	1965 (45.3%)	8.5	829 (19.1%)	Both (50.8%)	3509 (80.9%)	CS	Survey	Behaviour Problems Index, Zhang-Yeung Test of Achievement	Psychological well-being, cognitive development		Yes
Luo et al.^35^	China	1101	466 (42.3%)	14	822 (74.7%)		279 (25.3%)	Cohort	Survey	Mental Health Test, Resilience Youth Development Module	Mental health status, Resilience		Yes
Ma et al.^79^	China	15,623	7408 (48.4%)	15.1	5963 (38.9)		9660 (61.1)	CS	Survey	Loneliness scale, Resilience scale for Chinese Adolescents, Chinese version of Functional Assessment of Self-mutilation	Self-harm, suicide attempt, suicide ideation, loneliness, resilience		Yes
Man et al.^53^	China	2406	1191 (49.5%)	14.44	1309 (54.4%)	Both (56%)	1097 (45.6%)	CS	Survey	Scale of Mental Health for Chinese Middle-school Student	Depression, anxiety	Parent: 522 (39.9%)	Yes
Nguyen and Nguyen^70^	Vietnam	792	386 (48.74%)	12.65	439 (55.43)		353 (44.57%)	CS	Survey	SDQ	Emotional and behavioural problems, conduct problems, peer problems		Yes
Peng et al.^78^	China	15,232	7345 (48.2%)	15.18	3752 (24.6%)		11,480 (75.4%)	CS	Survey	Self-administered questionnaire	Internet addiction, Suicidal behaviours	Parents: 2146 (57.3%)	Yes
Qu et al.^40^	China	19711	9773 (49.6%)		7332 (37.2%)		12379 (62.8%)	CS	Interview	Chinese version of the Child Behaviour Checklist, Mini International Neuropsychiatric Interview	Psychological problem		Yes
Shao et al.^12^	China	1250	588 (47.0%)	11.22	635 (50.8%)	Both (29.44%)	615 (49.2%)	CS	Survey	Child Loneliness Scale, Depression Self-Rating Scale for Children, Cohesion Subscale of the Family Adaptation and Cohesion Evaluation Scales II	Depression, parent-child relationship		Yes
Shen et al.^54^	China	2283	1256 (55.0%)	14.22	1397 (61.2%)	Both (15.5%)	886 (38.8%)	CS	Survey	CDI, Screen for Child Anxiety Related Emotional Disorders	Depression, anxiety	Parent: 1185 (84.8%)	Yes
Shi et al.^36^	China	17635			8676 (49.2%)	Both (25.36%)	8959 (50.8%)	Cohort	Survey	Mental Health Test, Social Anxiety Scale for Children, Self-esteem Scale	Anxiety, self-esteem		Yes
Sukamdi & Wattie^29^	Indonesia	451	227 (50.33%)		207 (45.9%)	Both (54.1%)	244 (54.1%)	CS	Survey	SDQ	Health behaviour (smoking)	Parent: 192 (92.8%)	Yes
Tang (2016)^55^	China	1801	918 (50.916%)	12.6	351 (19.49%)	Father (12.2%)	1450 (80.51%)	CS	Survey	Standardized questionnaire	Depression		Yes
Tang et al.^41^	China	3346	1740 (52%)	13.9	1663 (49.7%)		1683 (50.3%)	CS	Interview	Standardized questionnaire	Emotional and behavioural problems, peer bullying, loneliness	Grandparent: 1367 (82.2%)	Yes
Tomsa & Jenaro^32^	Romania	326	158 (48.47%)	13.5	163 (50%)		163 (50%)	CS	Survey	State-Trait Anxiety Inventory for Children, short mood and feelings questionnaire, Anger Expression Scale for Children, Coping Strategies Checklist for Children	Anxiety		Yes
Turnip & Umami (2019)^30^	Indonesia	629	335 (53.2%)	13.5	359 (57.1%)		270 (42.9%)	CS	Survey	SDQ	Emotional and behavioural problem, conduct problems	Parent: 195 (54.3%)	Yes
Vanore (2015)^33^	Romania	1979	991 (50.1%)	10.8	471 (23.8%)	Father (11%)	1508 (76.2%)	CS	Survey	SDQ	Emotional and behavioural problem	Parent: 414 (88%)	Yes
Wang CDC et al.^83^	China	1236	557 (45.1%)	13.1				CS	Survey	SDQ	Emotional and behavioural problems, peer support, conduct problems, peer bullying, loneliness, conduct problems, health behaviours (smoking, alcohol)		Yes
Wang et al.^104^	China	1110	554 (49.9%)	14.4	561 (50.5%)	Both (18.4%)	549 (49.5%)	CS	Survey	The Adolescent Self-Harm Scale	Self-harm/ behavioural problems	Grandparents: 395 (70.4%)	Yes
Wang et al.^44^	China	4565	1981 (43.4%)	13	3136 (68.7%)		1429 (31.3%)	CS	Survey	SDQ, Youth Risk Behaviour Survey	Emotional and behavioural problems, peer support, conduct problems, smoking, alcohol		Yes
Wang et al.^74^	China	2186	956 (43.73%)	15.1	1026 (46.94)		1160 (43.06%)	CS	Survey	SDQ, self-injurious thoughts and behaviours (SITB), composite international diagnostic interview (CIDI)	Conduct problems, suicidal ideation, self-harm		Yes
Wang et al.^47^	China	124,357			22,855 (18.4%)		101,502 (81.62%)	CS	Survey	Patient Health Questionnaire (PHQ-2)	Self-harm, depression		Yes
Wang et al.^66^	China	1420	899 (46.9%)	12.04	792 (55.77%)		628 (44.23%)	CS	Survey	Cyber Victmin and Bullying Scale (CVBS)	Bullying		Yes
Wang F et al.^76^	China	1992		13.1	1259 (63.2%)		733 (36.8%)	CS	Survey	Standardized questionnaire	Emotional and behavioural problems, peer support, suicide ideation and attempt,		Yes
Wang JY et al.^56^	China	1347	641 (47.6%)	12.5	492 (36.5%)	Father (49.6%)	855 (63.5%)	CS	Survey	Patient Health Questionnaire-9 for depression, General Anxiety Disorder-7	Depression, anxiety, self-esteem	Parent: 412 (83.7%)	Yes
Wen & Lin (2011)^7^	China	635	314 (49.4%)	12.8	313 (49.3%)	Both (24.1%)	322 (50.7%)	CS	Survey	Standardized questionnaire	Psychological and behavioural problem	Grandparent: 228 (72.85%)	Yes
Wen et al.^85^	China	864	452 (52.3%)	14.1	387 (44.8%)	Both (57.1%)	477 (55.2%)	CS	Survey	Psychometric properties of positive youth development	Peer support, parent-child relationship, school support, academic pressure		Yes
Wen et al.^68^	China	776	411 (53%)	14.5	600 (77.3%)	Both (83.3%)	176 (22.7%)	CS	Survey	Childhood Trauma Questionnaire-Short Form, Conners Teacher Rating Scale	Emotional and behavioural problems, conduct problems	Grandparent: 495 (82.5%)	Yes
Wu et al.^57^	China	624	375 (60.1%)	12.5	162 (25.9%)		462 (74.1%)	CS	Survey	CES-DC	Depression		Yes
Wu et al.^37^	China	11,153	5632 (50.5%)	15.5				Cohort (0, 1, 2 year)	Survey	Hospital anxiety and Depression scale (HADS)	Depression, loneliness, emotional and behavioural problems		Yes
Wu et al.^80^	China	1175	607 (51.7%)	14.54	469 (39.9%)		706 (60.1%)	Longitudinal	Survey	UCLA loneliness scale, Social anxiety scale for children (SASC), mobile phone addiction index	Anxiety, loneliness, internet addiction		Yes
Xie et al.^62^	China	1957		11.93	1054 (53.86%)		903 (46.14%)	CS	Survey	Children’s Depression Inventory (CDI), Beck’s depression inventory (BDI), Olweus Bully or Victim Questionnaire	Self-harm, depression, suicidal ideation		Yes
Xing (2017)^67^	China	928	482 (51.9%)	14.2	401 (43.2%)	Father (68.7%)	527 (56.8%)	CS	Survey	Paediatric Quality of Life Questionnaire (PedsQLTM4.0)	-		Yes
Xu et al.^64^	China	534	283 (53%)	13.1	440 (82.4%)		94 (17.6%)	CS	Survey	Adolescent Psychological Adaptability Scale	Anxiety, academic pressure, loneliness, emotional and behavioural problems, conduct problem	Grandparents: 274 (62.3%)	Yes
Xu et al.^63^	China	1036	497 (48%)		380 (36.7%)		656 (63.3%)	CS	Survey		Depression, bullying, academic stress		Yes
Xu et al.^105^	China	1697	1131 (49.2%)	11.9	912 (38.7%)		785 (33.3%)	CS	Survey	Olweus Bully/ Victim Questionnaire (BVQ)	Bullying		Yes
Yan & Chen (2013)	China	225	107 (47.6%)	11.0	104 (46.2%)		121 (53.8%)	CS	Survey	House-Tree-People test	Psychological development		Yes
Yue et al.^58^	China	1328	677 (51%)		1036 (78%)	Both (22%)	292 (22%)	CS	Survey	CDI—Chinese version	Depression, self-esteem, parent-child relationship		Yes
Zhang et al.^59^	China	8353	9088 (49.4%)	7.58	13264 (72.1%)	Both (58.2%)	5132 (27.9%)	CS	Survey	CDI—Chinese version	Depression, peer support, parent-child relationship, school support, peer bullying		Yes
Zhang et al.^45^	China	4187	1980 (47.3%)	8.46	1470 (35.1%)		2717 (64.9%)	CS	Survey	SDQ	Emotional and behavioural problems, peer support, conduct problems, peer bullying, smoking, alcohol		Yes
Zhang et al.^88^	China	1,992	897 (45%)	13.1	1,724 (86.5%)		268 (13.5%)	CS	Survey	SDQ	Psychological well-being, cognitive development		Yes
Zhang et al.^106^	China	424	185 (43.6%)	13.9	209 (49.3%)		215 (50.7%)	CS	Survey		Conduct problem, Parent-child relationship, Loneliness		Yes
Zhao et al.^1^	China	2917	1383 (47.4%)	12.5	1695 (58.1%)		1222 (41.9%)	CS	Survey	Social Anxiety Scales for Children	Anxiety		Yes
Zhao et al.^65^	China	3538	1857 (52.5%)	12.4	2837 (80.2%)		701 (19.8%)	CS	Survey	SDQ	Emotional and behavioural problems, conduct problems, peer bullying	Grandparent: 1850 (65.2%)	Yes
Zhao et al.^46^	China	2102	984 (46.8%)	13.5	1423 (67.7%)	Both (17%)	679 (32.3%)	CS	Survey	CDI–Chinese version	Depression, academic pressure, loneliness, conduct problems		Yes
Zhao et al.^107^	China	322	111 (47.8%)	14.4	236 (73.3%)		86 (26.7%)	CS	Survey	Rosenberg self-esteem scale, social support rating scale	Self-esteem		Yes
Zhao et al.^61^	China	1704	844 (49.53%)	11.9	561 (32.92%)		1143 (67.08%)	CS	Survey	Children’s Depression Inventory (CDI), Beck’s depression inventory (BDI)	Suicidal ideation, depression		Yes
Zhang et al.^88^	China	2147	1,022 (47.6%)	13.9	742 (34.6%)		1405 (65.4%)	CS	Survey	Self-administered questionnaire	Life satisfaction, bullying		Yes
Zhou et al.^48^	China	5,026	1,763 (53.2%)	12.6	3,316 (67%)		1,636 (33%)	CS	Survey	SDQ	Emotional and behavioural problems		Yes
Zhou et al. (2021)	China	2360	1289 (54.6%)	15.0	1336 (56.6%)		1024 (43.39%)	CS	survey		Smoking, alcohol consumption		Yes

*CS* cross-sectional study, *SDQ* strengths and difficulties questionnaire, *CDI* children’s depression inventory, *CES-DC* centre of epidemiological studies depression scale for children^108–116^

### Outcome measurement

There was significant variation in the inclusion criteria and mental health assessment tools used for data collection across studies. Sixty-one studies data was collected using a questionnaire; four studies collected data by interviewing participants.^41–44^ Strengths and Difficulties questionnaire was the most commonly used survey.^29,31–33,36,37,45–49^

The total number of participants was 394,308 and the sample size of individual studies ranged from 225^35^ to 124,357.^50^ The estimated mean age of participants was 12.89 years, ranging from 6.51 years^51^ to 15.87.^52^ With 149,380 children identifying as ‘left-behind’, the LBC group accounted 37.9% of the study population.

### Effects of left-behind status on psychological wellbeing of children

#### Depression symptoms

**Fig. 2:**
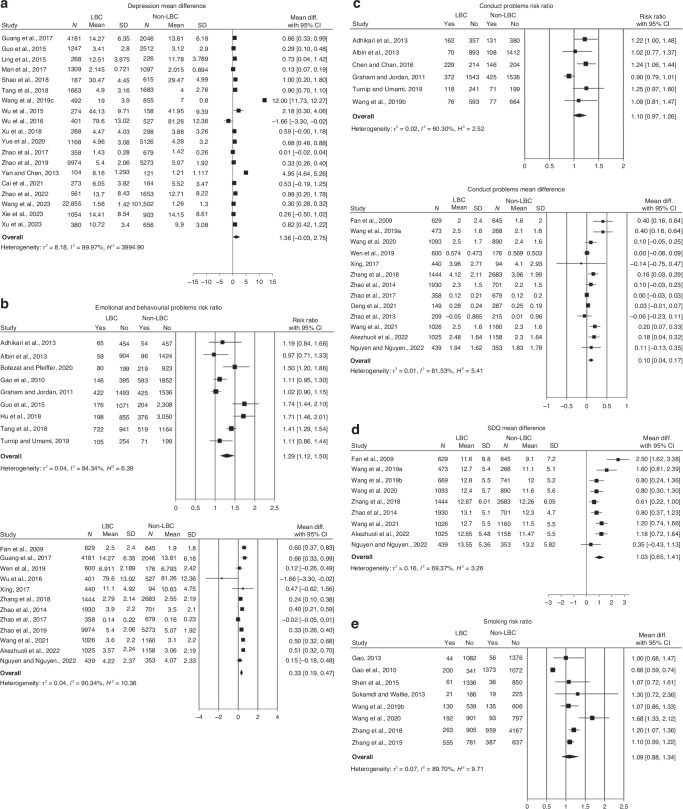
Forest plot for (**a**) Behavioural and emotional problems, (**b**) Conduct problems, (**c**) Strengths and difficulties questionnaire score, (**d**) Depression, (**e**) Smoking.

As presented in Table 2 and Fig. 2, nineteen studies with a total of 177,205 participants measured depressive symptoms.^12,40,44,49–51,53–66^ LBC had significantly greater depressive symptoms when compared to NLBC, with a mean difference of 1.362 (95% CI: 0.066 to 2.659; *p* < 0.05). Heterogeneity was statistically significant (I^2^ = 99.845%, *p* < 0.001).

**Table 2:** Result of all variable analysis of included studies in meta-analysis

Mean difference					Effect size		
Continuous variables	Studies (*n*)	MD (95% CI)	Q test	I^2^(%)	Effect size (95% CI)	Q test	I^2^(%)
Depression	19	1.362 (0.066, 2.659)*	8397.56***	3994.90	0.603 (-0.014, 1.220)	2192.13***	99.94
SDQ	9	1.128 (−17.786, 20.042)	0.01	0.00	0.002 (−0.029, 0.032)	0.01	0.03
Anxiety	9	1.471 (0.082, 2.860)*	1025.61***	99.97	0.919 (0.057, 1.781)*	9151.96***	99.83
Self−esteem	7	−1.107 (−2.602, 0.389)	2320.74***	99.93	−0.924 (−2.015, 0.167)	1614.73***	99.93
Peer Support	10	−0.503 (−1.225, 0.220)	2905.41***	99.78	−0.698 (−2.048, 0.652)	608.24***	99.96
Parent Supports	9	−0.628 (−1.378, 0.121)	212.78***	99.89	−0.139 (−0.264, −0.014)*	88.32***	95.90
School Supports	6	−0.060 (−0.135, 0.015)	24.02***	79.96	−0.074 (−0.160, 0.012)	25.60***	77.56
Loneliness	9	0.868 (−0.078, 1.815)	275.97***	99.88	0.131 (0.018, 0.245)*	42.85***	96.28
Behavorial and emotional problems	12	0.330 (0.191, 0.469)*	191.26***	90.34	0.116 (0.048, 0.185)*	51.66***	87.46
Conduct problems	13	0.108 (0.040, 0.176)*	43.98***	63.69	0.090 (0.060, 0.120)*	17.11	0.18
Peer Bullying	13	0.150 (0.060, 0.239)*	157.17***	88.61	0.079 (0.013, 0.145)*	111.62***	89.00
Risk ratio							
Categorical variables		RR (95% CI)	Q test	I^2^(%)			
Self−harm	5	2.323 (1.309, 4.121)*	103.58***	94.85			
Peer abuse and bullying	11	1.466 (1.180, 1.820)*	26.05**	77.79			
Loneliness	5	1.758 (0.998, 3.097)	55.35***	87.46			
Behavioural and emotional problem	9	1.803 (1.213, 2.679)*	50.54***	84.34			
Conduct problem	6	1.256 (0.847, 1.866)	14.65*	60.30			
Prolonged screen time and Internet addiction	4	1.535 (0.861, 2.742)	12.99**	79.58			
Drinking	6	1.866 (1.030, 3.381)*	126.2***	94.55			
Smoking	8	1.211 (0.679, 2.153)	83.26***	89.70			

*p < 0.05, **p < 0.01, ***p < 0.001

As presented in Table 3, subgroup analysis results found that younger LBC aged 6 to 12 had significantly worse depression level (MD = 1.281, 95% CI = 0.033 to 2.529, *p* < 0.05). No significant mean difference in depression scores between LBC and non-LBC is identified for children 13 years and older. Worse mean depression scores in LBC were only identified in studies that included current LBC (MD = 0.971, 95% CI = 0.163 to 1.779, *p* < 0.05). In terms of financial situation, children from a poorer household had significantly worse depression as compared to their NLBC counterparts (MD = 0.809, 95% CI = 0.590 to 1.027). Age of parental migration also influenced depression scores. Left-behind children whose parents migrated when they were under 7 years of age had significantly higher mean depression score compared to NLBC (MD = 0.656, 95% CI = 0.389, 0.924). However, parental migration when the child is 7 years or older did not significantly influence mean depression score in LBC.

**Table 3:** Subgroup analysis for continuous variables (depression, SDQ, emotional and behavioural problems, smoking, and peer bullying)

Subgroups	Studies (*n*)	Participants (*n*)	I^2^ (%)	Q-test	Mean difference (95% CI)
Depression					
Mean age of child (Years)					
6 to 12	7	15,668	98.42	644.66	1.281 (0.033, 2.529)*
$\ge$13	7	9106	99.99	7487.10	2.306 (−0.927, 5.540)
Left behind Status					
Current	11	159,381	1.813	880.44	0.971 (0.163, 1.779)*
Mixed	2	1469	1.895	5.12	0.883 (−1.202, 2.969)
Region of residence					
Urban	2	124,582	99.88	834.68	2.622 (−1.935, 7.179)
Rural	13	24,792	99.96	7523.29	1.594 (−0.147, 3.334)
Mixed	3	6752	62.20	5.46	0.466 (0.086, 0.846)
Mean GDP of province					
Above national standard	5	147,764	99.99	7143.47	2.777 (−1.746, 7.300)
Below national standard	7	10,257	90.60	65.53	0.455 (0.086, 0.824)*
Multiple provinces	5	12,294	89.25	52.75	0.510 (0.096, 0.924)*
Financial situation					
Average (>2k yuan)	4	12,370	99.98	7424.03	3.240 (−2.489, 8.968)
Poor (<2k yuan)	3	10,072	41.39	4.38	0.809 (0.590, 1.027)*
Age of child when parents migrated					
<7 years old	3	7307	0.00	0.09	0.656 (0.389, 0.924)*
$\ge$7 years old	2	1779	99.03	102.84	7.136 (−2.487, 16.759)
SDQ					
Mean age of child (Years)					
6 to 12	3	6193	90.95	16.47	1.121 (−0.163, 2.406)
$\ge$13	6	11,134	0.00	5.42	1.010 (0.803, 1.216)*
Left behind Status					
Current	6	12,660	30.34	7.30	0.862 (0.623, 1.100)*
Mixed	3	3998	81.90	11.50	1.580 (0.608, 2.552)*
Region of residence					
Rural	5	10,183	84.58	18.10	1.184 (0.547, 1.822)*
Mixed	3	6352	0.00	1.64	1.074 (0.800, 1.347)*
Parents migration status					
Mixed (one parent or both parents migrated)	7	12,958	78.50	20.05	1.003 (0.537, 1.469)*
Both parents migrated	2	4369	0.00	0.00	1.190 (0.863, 1.516)*
Main caregiver education level					
Junior high school or above	2	6110	0.00	0.35	0.681 (0.375, 0.987)*
Primary school or below	2	3905	91.31	11.51	1.605 (−0.059, 3.268)
Age of child when parents migrated					
<7 years old	2	2015	54.78	2.21*	2.029 (1.148, 2.909)*
$\ge$7 years old	2	3393	0.00	0.00	0.800 (0.427, 1.173)*
Emotional and behavioural problems					
Mean age of child (Years)					
6 to 12	4	12,420	74.07	11.59	0.404 (0.164, 0.644)*
$\ge$13	7	20,225	93.38	148.75	0.311 (0.134, 0.488)*
Left behind Status					
Current	7	33,393	56.25	13.12	0.383 (0.285, 0.481)*
Mixed	4	3621	85.46	28.07	0.246 (−0.103, 0.595)
Region of residence					
Rural	7	31,319	94.45	143.60	0.316 (0.137, 0.495)*
Mixed	3	4903	0.00	0.01	0.504 (0.374, 0.635)*
Main caregiver education level					
Junior high school or above	2	19,374	23.42	1.31	0.306 (0.229, 0.384)*
Primary school or below	2	3905	41.82	1.72	0.488 (0.293, 0.682)*
Smoking					
Mean age of child (Years)					
6 to 12	3	6193	40.04	3.52	0.207 (0.061, 0.353)*
$\ge$13	10	11,894	80.27	27.99	0.077 (0.012, 0.143)*
Left behind Status					
Current	6	12,343	17.60	6.56	0.127 (0.061, 0.194)*
Mixed	6	6345	90.06	22.37	0.134 (−0.023, 0.292)
Region of residence					
Rural	7	11,010	88.71	28.07	0.117 (−0.003, 0.237)
Mixed	4	6886	0.00	1.98	0.158 (0.078, 0.238)
Parents migration status					
Mixed (one parent or both parents migrated)	9	15,768	80.90	29.90	0.107 (0.017, 0.196)*
Both parents migrated	3	3805	74.16	9.04	0.121 (0.003, 0.238)*
Main caregiver education level					
Junior high school or above	2	6110	0.00	0.36	0.134 (0.036, 0.231)*
Primary school or below	2	3905	78.15	4.58	0.232 (−0.060, 0.524)
Peer Bullying					
Mean age of child (Years)					
6 to 12	4	12,420	65.60	7.66	0.226 (0.056, 0.395)
$\ge$13	5	22,988	0.00	0.62	0.166 (0.128, 0.204)*
Left behind Status					
Current	8	25,429	70.71	22.92	0.141 (0.063, 0.218)*
Mixed	2	2015	21.28	1.27	0.302 (0.106, 0.498)*
Region of residence					
Rural	7	36,541	93.47	128.57	0.172 (0.032, 0.313)*
Mixed	3	5405	77.46	9.91	0.094 (−0.056, 0.245)
Parents migration status					
Mixed (one parent or both parents migrated)	11	25,575	91.11	151.09	0.147 (0.042, 0.253)*
Both parents migrated	2	4369	0.00	0.24	0.175 (0.074, 0.276)*
Main caregiver education level					
Junior high school or above	3	25,668	97.72	106.46	0.064 (−0.151, 0.279)
Primary school or below	2	3905	47.73	1.91	0.276 (0.086, 0.466)*
Financial situation					
Average (>2k yuan)	4	12,369	24.18	3.58	0.255 (0.144, 0.366)*
Poor (<2k yuan)	2	8925	95.00	19.98	0.018 (−0.325, 0.360)

*p < 0.05, **p < 0.01, ***p < 0.001

#### Anxiety symptoms

Nine studies with a total of 29,284 participants investigated anxiety in children^35,39,56,57,59,67–69^ LBC had significantly greater anxiety than NLBC, with a mean difference of 1.471 (95% CI: 0.082 to 2.860, *p* < 0.05) (See Table 2). A small significant effect size of 0.919 (95% CI: 0.057 to 1.781, *p* < 0.05) was also identified. Heterogeneity for mean difference (I^2^ = 99.97%, *p* < 0.001) and effect size were statistically significant (I^2^ = 99.83%, *p* < 0.001).

#### Emotional and behavioural problems

Twenty-one with 61,951 participants investigated emotional and behavioural problems in LBC.^1,29,31,33,34,37,40,45,47,49,53,54,62,68,70–76^ Twelve studies measured behavioural and emotional problems as a continuous variable, while nine studies measured behavioural and emotional problems as a categorical variable. As presented in Table 2, LBC had more emotional and behavioural problems than NLBC, with a mean difference of 0.330 (95% CI = 0.191 to 0.469; *p* < 0.05) and effect size of 0.116 (95% CI = 0.048 to 0.185). Heterogeneity for mean difference (I^2^ = 90.34%, *p* < 0.05) was statistically significant. Similar results were identified in studies that measured emotional and behavioural problems as a categorical variable. The risk of having behavioural and emotional problems was also greater in LBC (RR = 1.803, 95% CI = 1.213 to 2.679) than NLBC.

Thirteen studies with 37,942 participants investigated conduct problems as a continuous variable,^1,40,45,49,53,62,68,70–73,77^ and six studies with 24,009 participants analysed it as a categorical variable.^29,31,33,37,78,79^ As a continuous variable, the prevalence of conduct problems was significantly greater in LBC than NLBC (MD: 0.108, 95% CI: 0.040 to 0.176; *p* < 0.05), but its effect size was minimal at 0.090 (95% CI: 0.060 to 0.120; p < 0.05). As a categorical variable, the risk of having conduct problems was not significantly greater in LBC compared to NLBC (RR: 1.256, 95% CI: 0.847 to 1.866; *p* > 0.05). Heterogeneity was only significant for mean difference (I^2^ = 63.69%, *p* < 0.001).

Subgroup analysis results for categorical variables were reported in Table 4. Younger LBC aged 6 to 12 years (RR = 1.439, 95% CI = 1.014 to 2.043, *p* < 0.05) were at greater risks of having behavioural and emotional problems as compared to older LBC 13 years and over (RR = 1.234, 95% CI = 1.063 to 1.433, *p* < 0.05). Similarly, a greater mean difference in emotional and behavioural problem scores was identified between younger LBC and NLBC aged 6 to 12 (MD = 0.404, 95% CI = 0.164 to 0.644, *p* < 0.05) as compared to older children 13 years or more (MD = 0.311, 95% CI = 0.134 to 0.488, *p* < 0.05). Based on region of residence, only studies that included LBC from rural regions (RR = 1.259, 95% CI = 1.063, 1.492, *p* < 0.05) had a greater risk of having behavioural and emotional problems as compared to their NLBC counterparts.

**Table 4:** Subgroup analysis for categorical outcomes (self-harm, smoking, behavioural and emotional problems)

Subgroups	Studies (*n*)	Participants (*n*)	I^2^ (%)	Q-test	RR^Risk Ratio^ difference (95% CI)
Self Harm					
Mean age of child (Years)					
6 to 12	4	5867	79.24	16.52	1.268 (1.046, 1.538)*
$\ge$13	5	22,612	17.18	5.15	1.168 (1.122, 1.216)*
Region of residence					
Rural	7	26,581	68.49	13.45**	1.130 (1.045, 1.222)*
Mixed	2	3654	0.00	0.003	1.479 (1.281, 1.709)*
Mean GDP of province					
Below national standard	2	5387	77.83	4.51	1.587 (1.173, 2.148)*
Multiple provinces	2	17,269	58.21	2.39	1.236 (0.953, 1.603)
Parents migration status					
One parent migrated only	4	21,604	70.11	11.84	1.057 (0.918, 1.216)
Mixed (one parent or both parents migrated)	7	24,004	56.74	13.95	1.255 (1.144, 1.376)*
Main caregiver					
Parents	4	18,875	30.41	4.77	1.108 (0.983, 1.248)
Grandparents	2	4139	86.89	7.51	1.106 (0.914, 1.339)
Smoking					
Mean age of child (Years)					
6 to 12	2	6745	0.00	0.07	1.207 (1.073, 1.359)*
$\ge$13	6	17,514	91.30	71.65	1.049 (0.809, 1.360)
Left behind Status					
Current	4	13,050	94.43	62.16	0.980 (0.747, 1.286)
Mixed	2	4541	80.44	5.11	1.328 (0.799, 2.206)
Region of residence					
Urban	1	2283			1.075 (0.718, 1.608)
Rural	4	15,608	90.96	53.06	0.952 (0.715, 1.267)
Mixed	2	4343	90.81	10.88	1.341 (0.885, 2.033)
Mean GDP of province					
Above national standard	3	6954	84.87	17.12	0.868 (0.627, 1.203)
Below national standard	2	4266	71.90	3.56	1.387 (0.899, 2.142)
Multiple province	2	8654	21.57	1.27	1.143 (1.047, 1.248)
Parents migration status					
One parent migrated only	2	4541	80.44	5.11	1.328 (0.799, 2.206)
Mixed (one parent or both parents migrated)	6	15,784	89.33	63.34	1.014 (0.818, 1.257)
Mother’s education level					
Junior high school or above	4	13,195	81.76	11.70	1.230 (1.008, 1.502)*
Primary school or below	2	5269	80.93	5.24	0.808 (0.503, 1.299)
Financial situation					
Average (>2k yuan)	3	9687	81.40	8.73	1.283 (0.999, 1.648)
Poor (<2k yuan)	2	4918	0.00	0.22	1.093 (0.991, 1.205)
Emotional and behavioural problems					
Mean age of child (Years)					
6 to 12	3	12,114	93.62	37.03	1.439 (1.014, 2.043)*
$\ge$13	5	10,865	69.16	13.16	1.234 (1.063, 1.433)*
Region of residence					
Rural	6	14,953	79.94	22.57	1.259 (1.063, 1.492)*
Mixed	2	7635	95.45	21.98	1.322 (0.782, 2.236)
Parents migration status					
One parent migrated only	3	6859	62.65	5.64	1.223 (0.972, 1.537)
Mixed (one parent or both parents migrated)	5	16,950	86.96	43.65	1.330 (1.094, 1.495)*
Mother’s education level					
Junior high school or above	3	6210	48.00	3.90	1.512 (1.248, 1.833)*
Primary school or below	3	8094	86.57	16.85	1.294 (0.969, 1.728)
Country					
China	4	13,294	88.74	19.62	1.297 (1.027, 1.638)*
Other Asian countries	4	9294	82.97	22.17	1.236 (0.961, 1.590)

*p < 0.05, **p < 0.01

Similar subgroup analyses results for conduct problems as a continuous variable were found. At younger ages (MD = 0.207, 95% CI = 0.061 to 0.353, *p* < 0.05), a greater mean difference in conduct problems between LBC and NLBC was identified than studies with children with a mean age 13 years or more (MD = 0.077, 95% CI = 0.012 to 0.143, *p* < 0.05). Only studies that included current LBC resulted in significant mean differences in conduct problems between LBC and NLBC (MD = 0.127, 95% CI = 0.061, 0.194, *p* < 0.05).

#### Loneliness

Nine studies with a total of 50,495 participants explored loneliness as a continuous variable,^10,40,49,68,70,80–83^ and 5 studies with 22,303 participants measured loneliness as a categorical variable.^19,30,44,74,79^ No significant mean difference for loneliness experienced was found between LBC and non-LBC when measured as a continuous variable (MD: 0.868, 95% CI = −0.078 to 1.815; *p* > 0.05). However, a minor significant effect size of 0.131 (95% CI = 0.018 to 0.245, *p* < 0.05) was identified.

#### Self-harm

Five studies with a total of 147,013 participants explored suicide ideation and attempts,^19,59,74,84^. LBC had 2.323 times the risk (95% CI: 1.309 to 4.121; *p* < 0.05) of committing self-harm compared to NLBC. The heterogeneity was statistically significant (I^2^ = 94.85%, *p* < 0.001).

#### Self-esteem

Seven studies with a total of 31,982 participants measured self-esteem.^39,44,63,67,85–87^ No significant differences in self-esteem were found between LBC and NLBC.

#### Child support—peer, parent, and school

Nine studies with a total of 33,704 participants investigated parent-child relationships in the form of parental support.^10,12,49,51,54,61,62,85,88^ Parental support in this study was measured by parental responsiveness and sensitivity to needs of the child. Some scales used across different studies included the Parent-Child Relationship Scale (PCRS) and Family Adaptation and Cohesion Evaluation Scales.^89,90^ Parental support was significantly worse in LBC than NLBC with an effect size of −0.139 (95% CI = −0.264, −0.014, *p* < 0.05). The heterogeneity for effect size (I^2^ = 95.90%, *p* < 0.001) was statistically significant.

Ten studies with a total of 34,187 participants measured peer support,^21,42,51,61,62,81,86–88,91^ while six studies with a total of 14,567 participants measured school support.^42,51,54,61,88,92^ No significant differences in peer support and school support were found between LBC and NLBC.

#### Peer bullying

Eleven studies with a total of 36202 participants explored peer bullying as a categorical variable.^19,29,34,37,44,66,72,73,77–79^ While thirteen studies measured peer bullying as a continuous variable.^1,45,49,51,53,61,62,66,72,73,77,86^ LBC were 1.466 times more likely (95% CI = 1.180 to 1.820; *p* < 0.05) to have experienced peer bullying compared to NLBC. As a continuous variable, LBC were significantly more likely to have experienced peer bullying compared to NLBC (MD: 0.150, 95% CI: 0.060 to 0.239; *p* < 0.05). The heterogeneity was statistically significant (I^2^ = 88.61%, *p* < 0.001).

Subgroup analysis revealed that significant mean differences in peer bullying between LBC and NLBC were only found in children aged 13 years or older (MD = 0166, 95% CI = 0.128 to 0.204, *p* < 0.05). Significant mean differences in peer bullying between LBC and NLBC were only identified from studies that sampled children residing in rural regions (MD = 0.172, 95% CI = 0.032 to 0.313, *p* < 0.05).

#### Prolonged screen time and Internet addiction

Four studies with a total of 21,940 measured prolonged screen time and internet addiction.^57,63,74,93^ No significant difference was identified in prolonged screen time and internet addiction between LBC and NLBC.

#### Alcohol consumption and smoking

Six studies with a total of 31,666 participants investigated past attempts at drinking alcohol.^41,62,74,86,91^ LBC had 1.866 times the risk (95% CI = 1.030 to 3.381; *p* < 0.05) of having drunk alcohol previously compared to NLBC. The heterogeneity for smoking was statistically significant (I^2^ = 89.70%, *p* < 0.001). Although the risk for past attempts for drinking was significantly higher in LBC, no significant difference in risk for smoking was found between LBC and non-LBC.

Subgroup analysis revealed that LBC aged 6 to 12 were more likely to attempt smoking (RR = 1.207, 95% CI = 1.073 to 1.359, *p* < 0.05). No significant difference in attempt for smoking was identified for LBC and NLBC 13 years or older (RR = 1.049, 95% CI = 0.809 to 1.360, *p* < 0.05).

#### Publication bias

Egger test results in appendix 2 suggest publication bias is minimal for all outcome variables except parental support (Egger’s value = −5.071, *p* = 0.0007), loneliness (Egger’s value = 6.454, *p* = 0.006) and conduct problems (Egger’s value = 1.809, *p* = 0.0009). Sensitivity analysis was done on these variables by removing one study per time, the cumulative analysis suggested that the results are not due to any single study.

## Discussion

Compared to controls that were not left behind, LBC was found to have more depression, anxiety, behavioural and emotional problems, conduct problems, self-harm, loneliness, peer bullying, and smoking and alcohol consumption.

Worse mental health in LBC may be explained by poor socio-emotional development at younger ages due to poor support from parents and peers, along with negative peer interactions.^94^ Lack of communication and contact from parents often lead to disruptions in parent-child attachment and greater feelings of loneliness amongst LBC. Disrupted parent-child attachment has been associated with worse emotional coping and depression.^14^ Similar results were also identified in the current study with LBC being at greater risk of having depression, behavioural and emotional problems, conduct problems, committing self-harm, and engaging in risk behaviours. While secure attachments allow for health socio-emotional development, equipping children with better social skills and coping strategies, disturbances in parent-child attachment are associated with reduced emotional resilience and poor emotional coping against psychological stress.^14^ Lack of healthy emotional coping and resilience against psychological stress predisposes LBC to greater risks of emotional and behavioural problems, poor mental health outcomes and engagement in risk behaviours.

Our findings of poor parental support in LBC and its association with more emotional and behavioural problems add value to existing knowledge, indicating the importance of parental support as a positive resilient factor in forming secure attachments to reach healthy socioemotional development as described in the resilience framework.^95–97^ Findings from our study supported results of previous studies that suggested an absence of parental care and the lack of adult models to demonstrate healthy emotional and behavioural patterns compromise the mental health of children and lead to more unsafe practices.^98–100^ Hence, poor socio-emotional development can increase the risk of engagement in risk behaviours such as smoking and drinking as an unhealthy emotional coping strategy against psychological stress. Lack of communication and distance between parents and LBC may lead to delayed and inappropriate responses to their child’s needs, which may further aggravate feelings of loneliness and isolation in LBC.^101^ Results from previous and the current study consistently suggest the importance of parents providing emotional and behavioural support and adult role models guiding children.^100^

The influence of parent-child attachment, family dynamics and parenting practices on the development of healthy emotional and behavioural regulation in children has been well established.^15^ Young age at separation for LBC acts as another contributing risk factor for poor mental health outcomes. Subgroup analysis revealed that studies with a younger LBC (mean age between 6 to 12 years) were at greater risks of poor mental health outcomes, including having higher mean score in depression, behavioural, and emotional problems, smoking attempts, conduct problems, and self-harm. This confirmed that parental migration at younger ages has a more significant influence on children. Although previous studies have found significantly worse mental health in LBC across children of all ages, majority of the studies and reviews have indicated worse anxiety and depression amongst LBC separated with their parents at younger ages.^27,28^ Young children are especially sensitive to the detrimental effects of disturbed family dynamics and absence of parental figures as rapid social and emotional development occurs in early childhood.^13^ However, prolonged parental separation at any age can have significant long-term detrimental effects on child socioemotional development and mental health.^14,26–28^

Negative peer interactions in the form of increased peer bullying may also explain worse mental health outcome in LBC. Poor socio-emotional development that accumulates from a younger age can negatively influence a child’s peer interactions at school. In the current study, we found that LBC were at greater risks of peer bullying victimization as compared to their counterparts that were not left behind. This aligns with previous studies that found a greater prevalence of bullying in children and adolescents with emotional, behavioural and conduct problems.^102^ Children with emotional, behavioural, and conduct problems may have difficulties in behavioural and emotional self-regulation, leading to destructive tantrums and outbursts of temper loss, hindering their ability to engage in social opportunities to form friendships at school.^96^ Bullying victimization presents as another psychological stressor linked to anxiety, depression and suicidality amongst children and adolescents.^103^ Disturbances in family dynamics at home due to prolonged separation from parents coupled with peer bullying at school can severely hurt the mental health of LBC.

In addition to prolonged and early parental separation, LBC are often left-behind in rural regions with worse structural and environmental supporting factors, including worse access to mental health and social services in conjunction with families poor financial situation and low income.^95^ In our study, we found that LBC residing in rural regions had significantly worse emotional and behavioural problems, lower SDQ scores and experienced more peer bullying than non-LBC, while no significant differences were identified between LBC and non-LBC in children residing in urban regions. Previous studies have identified multiple shortcomings in mental health services in rural China.^79^ This included a lack of available mental health services in rural towns and villages and a lack of mental health training in teachers, which prevents them from identifying signs of mental illness in children and adolescents.^79^

To mitigate adverse effects of being left behind, different promising interventions to improve psychological resilience have been implemented and researched. Interventions including group psychological services aimed to help LBCs develop emotional management strategies and improve psychological capital qualities such as self-efficacy, optimism, and hope have significantly improved mental health in LBC.^46,104^ Furthermore, the importance of a supportive school environment through peer and teacher support also appeared to be positive resilient factor to improve mental health and academic achievement.^105,106^

One limitation of the study was that most included studies were conducted in China and Southeast Asia. Thus, while the author acknowledges that cultural factors may influence resilience, this could not be analysed in the current study. Comparisons between LBC in low-to-middle-income countries and high-income countries also could not be made due to the lack of studies from different countries limiting the generalisability of the study.

## Conclusions

Prolonged parental separation negatively influenced mental health, especially in younger children between the ages 6 to 12. As improving household income is the primary reason prompting parental migration from rural regions into urban cities, policies to reduce inequalities in job opportunities are required. For current LBC, teachers and peers play an important role in providing social support for LBC. Furthermore, timely support targeted towards strengthening resilience factors including emotional management, interpersonal relationships, stress management and self-esteem development are required to improve the mental health of LBC.

## Supplementary information

Supplementary information

Supplementary information

## Data Availability

Extracted data will be made available upon reasonable request.
