# Supplementary material for: Urbanisation and mental health in left-behind children: systematic review and meta-analysis using resilience framework
Source: Pediatr Res. 2025 Feb 5;98(3):819–38. doi: 10.1038/s41390-025-03894-5 (PMC12507658; doi:10.1038/s41390-025-03894-5)
Supplement: Supplementary file 1 — Supplementary information [file 41390_2025_3894_MOESM1_ESM.pdf]

Appendix 1:

The following key terms were searched in MEDLINE, EMBASE, The Cochrane Library, Scopus, PsycINFO, and Web of Science: (left behind child*OR left behind adolescents OR parental migration) AND (self harm OR self injury OR suicid* OR resilience OR self-esteem OR bully* OR victimi* OR study OR academic OR friend* OR peer* support* OR 'life events' OR parent-child OR communication OR health-related behavior OR smok* OR alcohol OR aggressi* OR violen* OR emotional and behavioural problems OR depress*).

Appendix 2: Egger regression assessment of publication bias

Variable	Egger test	P
Depression	-1.384 (-7.088, 4.320)	0.615
SDQ	3.411 (-0.771, 7.593)	0.095
Anxiety	2.714 (-5.226, 10.654)	0.446
Self-esteem	1.329 (-9.168, 11.825)	0.758
Peer Support	-7.245 (-19.711, 5.220)	0.217
Parent Supports	-5.071 (-7.168, -2.974)	0.0007
School supports	0.164 (-5.573, 5.901)	0.941
Loneliness	6.454 (2.516, 10.391)	0.006
Conduct Problems	1.809 (0.929, 2.690)	0.0009
Peer Bullying	2.483 (-0.044, 5.009)	0.053
